# Time-Lagged Correlation Analysis of Shellfish Toxicity Reveals Predictive Links to Adjacent Areas, Species, and Environmental Conditions

**DOI:** 10.3390/toxins14100679

**Published:** 2022-09-30

**Authors:** André Patrício, Marta B. Lopes, Pedro Reis Costa, Rafael S. Costa, Rui Henriques, Susana Vinga

**Affiliations:** 1 INESC-ID—Instituto de Engenharia de Sistemas e Computadores-Investigação e Desenvolvimento, Instituto Superior Técnico, Universidade de Lisboa, 1000-029 Lisbon, Portugal; 2 NOVA Laboratory for Computer Science and Informatics (NOVA LINCS), FCT NOVA, 2829-516 Caparica, Portugal; 3 Center for Mathematics and Applications (NovaMath), FCT NOVA, 2829-516 Caparica, Portugal; 4 IPMA—Instituto Português do Mar e da Atmosfera, 1495-006 Lisboa, Portugal; 5 CCMAR—Centro de Ciências do Mar, University of Algarve, 8005-139 Faro, Portugal; 6 S2AQUA—Collaborative Laboratory, Association for a Sustainable and Smart Aquaculture, 8700-194 Olhão, Portugal; 7 LAQV-REQUIMTE—Associated Laboratory for Green Chemistry, Department of Chemistry, NOVA School of Science and Technology, Universidade NOVA de Lisboa, 2829-516 Caparica, Portugal

**Keywords:** toxicity, shellfish, DSP, Portuguese coast, correlation analysis

## Abstract

Diarrhetic Shellfish Poisoning (DSP) is an acute intoxication caused by the consumption of contaminated shellfish, which is common in many regions of the world. To safeguard human health, most countries implement programs focused on the surveillance of toxic phytoplankton abundance and shellfish toxicity levels, an effort that can be complemented by a deeper understanding of the underlying phenomena. In this work, we identify patterns of seasonality in shellfish toxicity across the Portuguese coast and analyse time-lagged correlations between this toxicity and various potential risk factors. We extend the understanding of these relations through the introduction of temporal lags, allowing the analysis of time series at different points in time and the study of the predictive power of the tested variables. This study confirms previous findings about toxicity seasonality patterns on the Portuguese coast and provides further quantitative data about the relations between shellfish toxicity and geographical location, shellfish species, toxic phytoplankton abundances, and environmental conditions. Furthermore, multiple pairs of areas and shellfish species are identified as having correlations high enough to allow for a predictive analysis. These results represent the first step towards understanding the dynamics of DSP toxicity in Portuguese shellfish producing areas, such as temporal and spatial variability, and towards the development of a shellfish safety forecasting system.

## 1. Introduction

Diarrhetic Shellfish Poisoning (DSP) is one of the most frequent poisonings caused by the ingestion of shellfish, potentially resulting in symptoms characteristic of severe gastrointestinal illness, such as nausea, vomiting, abdominal pain, and diarrhoea. This condition is caused by marine toxins okadaic acid (OA) and dinophysistoxins (DTX) [1,2], which are naturally produced by certain dinoflagellate species, such as *Dinophysis* spp. and *Prorocentrum* spp. Since shellfish are filter feeders with high filtration rates, when these toxin-producing dinoflagellates reach high cell abundance in the marine environment, shellfish will rapidly accumulate toxins in their tissues and become toxic for humans to consume.

Due to the nature of the symptoms caused by DSP, cases of this poisoning can easily go undetected and be mistaken for other gastrointestinal disorders, especially since most cases are diagnosed based on symptoms and no confirmatory testing is usually performed. The first reported outbreaks of DSP occurred in Japan in 1976 [3], while in Portugal, the first identified outbreak took place in 1998 [4]. Following the identification of the effects of these harmful toxins, multiple policies have been issued to protect the consumer from their adverse outcomes. Since 1991, the European Commission has enacted legislation that requires member state authorities to control quantities of DSP toxins in the edible parts of molluscs [5]. Since 2002, it further introduced: (i) legal limits for the concentration of various biotoxins in shellfish, 160 μg OA eq. kg^−1^ for DSP toxins [6]; (ii) the concept of indicator species, a species that has the highest rate of toxin accumulation and is therefore used as the control for that toxin/area [7], and (iii) weekly sampling for testing purposes [7] (regulation 854/2004 [7] is no longer in force; it has since been replaced by regulation 2019/627 [8]). As successful as these monitoring-based policies are at safeguarding consumers, they do not provide producers with an early warning fundamental to anticipate toxicity peaks and subsequent closures. Monitoring systems in Andalusia [9] and Galicia [10] further highlight the need to incorporate contextual variables, such as phytoplankton concentrations and oceanographic conditions, to aid risk assessment.

A fundamental additional step to these regulations is the thorough understanding of how other contextual variables, such as geographical location and meteorological conditions, interact and ultimately affect the levels of toxicity in shellfish. As the strength of the association of these contextual variables with shellfish toxicity is still arguably unknown, in this study, they are referred to as potential risk factors, or simply risk factors. Through the knowledge about the associations of toxins with risk factors, we can better predict how the levels of toxicity change and implement appropriate systems to anticipate the prohibition of shellfish harvesting.

Multiple studies have tackled the topic of toxins accumulation in shellfish and how it affects humans. Most studies are retrospective and look at patterns in shellfish toxicity levels [11,12]. Another common focus is the investigation of the relationship between the levels of toxin-producer phytoplankton and the toxicity found in shellfish [13], complemented by the study of how the concentration of phytoplankton is influenced by other variables such as meteorological conditions and nutrient availability [14,15]. Analysis of these types of patterns and relations is essential to better understand external factors that may influence shellfish toxicity and, consequently, to allow us to effectively intervene when necessary.

A possible extension of these advances is the development of predictive machine learning algorithms that can forecast the shellfish toxicity levels, and in this way prevent the consumption of contaminated shellfish while supporting the management of the production areas. Multiple contributions have been made to reach this goal [16]. In the domain of Bayesian methods, we have the work of Wang et al. [17], which uses Bayesian Networks to model DSP contamination in shellfish. Velo-Suárez and Gutiérrez-Estrada [18] applied a Neural Network to predict Harmful Algal Blooms (HABs) based on weekly data of phytoplankton concentration. Simpler models have also shown good results. Schmidt et al. [19] used Generalized Linear Models to predict variations in DSP toxicity in shellfish and Raine et al. [20] proposed a predictive model to estimate HABs based on the impact of wind-driven water exchanges in phytoplankton.

Regarding the analysis of temporal patterns, past research indicates that the concentration of DSP toxins tends to follow a seasonal pattern [21,22,23], a circumstance also found in Portugal [24]. We aim to corroborate this finding and expand this analysis with the addition of the annual trend and production area location components. Concerning the relations between risk factors and DSP toxicity, there is evidence that numerous variables influence toxicity values, namely spatial variation [21,25], phytoplankton concentration [26,27], and water temperature [15,28]. Thus, we studied how various risk factors relate to each other at different time points, making it possible to identify variables sufficiently correlated for use in a predictive analysis of DSP toxicity.

Accordingly, the present work advances the stated goals through two main contributions. First, we present an analysis of temporal patterns of DSP toxicity, particularly the seasonality and trend, aiming to better understand and predict peaks of toxicity. Second, we study the correlations between the DSP toxicity levels in shellfish and multiple variables, namely: (i) geographical location; (ii) shellfish species; (iii) toxic phytoplankton cell counts; and (iv) oceanographic and meteorological conditions. Through correlation analysis, we assess how these variables can be used to predict toxicity levels. Additionally, we extend this association analysis to include time lags, enabling the understanding of how a given variable correlates with the future values of another variable of interest. Although the present analysis used data collected across the Portuguese coast, the methodology presented here is data independent and can be easily extended to other regions and relevant variables.

## 2. Materials and Methods

### 2.1. Data Sources

**Figure 1:**
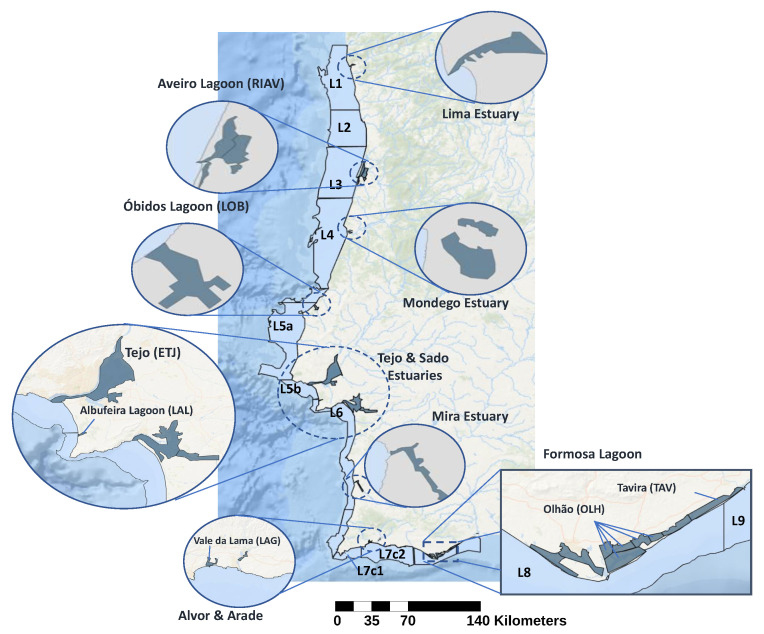
Shellfish production areas on the Portuguese coast as defined by IPMA (adapted from IPMA [31]).

To perform the analysis of the relations between toxicity levels in shellfish and other variables, we used three data sources spanning a total of 312 weeks, each representing a specific focus of our study. The collected data represent a total of 43 shellfish production areas, separate geographical locations defined by the Portuguese Institute for Sea and Atmosphere (IPMA) (available at www.ipma.pt/pt/bivalves/docs/files/Limites_ZDP_Litorais_2020.pdf and www.ipma.pt/pt/bivalves/docs/files/Limites_ZDP_Estuarino-Lagunares_2020.pdf (accessed on 29 July 2022), based on Despacho N.° 4362/2020 on April 9 of Diário da Républica [29]). These areas can be visualized in Figure 1, where each named area can include multiple sub-areas (e.g., RIAV includes the areas RIAV1, RIAV2, RIAV3, and RIAV4). The first dataset contains measurements of toxin levels in various shellfish species and production areas. The different variables are described in Table 1a. We analysed 4 of the 23 commercially harvested species, focusing on the most common ones to ensure that enough data were available to produce robust results. These species are mussel (*Mytilus galloprovincialis*), cockle (*Cerastoderma edule*), surf clam (*Spisula solida*), and Donax clam (*Donax trunculus*). The second dataset corresponds to measurements of toxic phytoplankton cell counts in the production areas (Table 1b). The last dataset consists of oceanographic and meteorological conditions recorded daily by IPMA and Copernicus [30] in the various production areas. The monitored variables are listed in Table 1c.

### 2.2. Preprocessing and Data Preparation

When dealing with multivariate time series data, it is important to have a defined periodicity consistent across the whole analysis. As such, all the datasets were resampled to a weekly representation using the mean estimator. This granularity was chosen because the sampling of phytoplankton and determination of shellfish toxicity roughly followed this periodicity, although the data often presented inconsistencies, e.g., multiple measurements in the same week and consecutive weeks without measurements.

After resampling and removal of outliers, the data presented some irregularities in the form of missing values in certain weeks. To address these irregularities, imputation techniques were applied. The imputation process used is completely automated. It selects the best imputation method and corresponding hyperparameters for each time series variable, as reported by Sousa et al. [32]. To ensure that the resulting data are not overly artificial, a minimum number of samples was defined, and so, only time series with at least 240 weekly measurements out of the 312 total weeks (from 5 January 2015 to 29 December 2020) were considered for imputation and further analysis.

The developed imputation algorithm starts by creating multiple copies of each time series and generating artificial missing values for each copy. These time series were then imputed using various methods (listed by Sousa et al. [32]). In each method, we applied Bayesian Optimization [33] to find the best hyperparameterization. Finally, model fitness was evaluated by determining the Root Mean Squared Error (RMSE),

(1) $$RMSE=\sqrt{\frac{\sum_{i=1}^{N}{\left(x_{i}-{\hat{x}}_{i}\right)}^{2}}{N}},$$

between the original time series, $x=(x_{1},x_{2},...,x_{n})$, and the generated copies with artificial missing values and subsequent imputation, $\hat{x}$. The best method and corresponding hyperparameterization were then selected as the ones to be used for that specific time series.

### 2.3. Seasonality-Trend Analysis

In order to better understand the major temporal determinants of shellfish toxicity, we analysed two components of the time series data: seasonality and trend. The seasonality of a time series represents periodic and predictable changes that occur at specific times every year, caused by some type of cyclic occurrence. This seasonality could correspond to an increase in shellfish toxicity at regular intervals, such as weekly, monthly, or quarterly. The trend of a time series expresses a long-term increase or decrease in its values. In this context, a trend could be the steady decrease in overall toxicity values across the years. These components are evaluated in two scenarios: the same species (mussel) in different production areas and different species in the same production area.

We further extend the analysis of the seasonality and trend components to different geographical areas by taking into account their geographical location on the Portuguese coast. In this geographical analysis, we grouped the production areas into three regions: northwest, southwest, and south. Using the map in Figure 1 as a reference, the northwest region encompasses the areas on the west coast above L4, the southwest region covers L4 and the areas below it, and the south region corresponds to the south coast of Portugal.

Production areas were chosen for this exploratory analysis based on being sufficiently regular and with frequent peaks of toxicity. The selected areas were, from north to south, L2, RIAV1, RIAV2, LOB, L5b, ETJ1, L7c1, LAG, OLH2, and L9.

For the analysis of the seasonality and trend components, we averaged the toxicity values by month and year, respectively. This approach was chosen in preference to a statistical decomposition of the various components [34] because our goal is to identify the temporal patterns of toxicity variation, which result from the action of many interconnected components.

### 2.4. Correlation Analysis

The focus of our analysis is to assess the impact of various variables on shellfish DSP toxicity, namely, how this variable is affected by geographical location, shellfish species, toxic phytoplankton abundance, and environmental conditions. This analysis aimed to identify the associations between these variables and toxicity levels using pairwise correlation analysis. We employed two methods: the Pearson correlation [35], and the Detrended Partial-Cross-Correlation Analysis (DPCCA) coefficient [36]. The Pearson correlation is calculated between two time series x and y,

(2) $$r=\frac{\sum \left(x_{i}-\bar{x}\right)\left(y_{i}-\bar{y}\right)}{\sqrt{\sum {\left(x_{i}-\bar{x}\right)}^{2}\sum {\left(y_{i}-\bar{y}\right)}^{2}}},$$

where $\bar{x}$ represents the mean of time series x. The DPCCA coefficient is an extension of the Detrended Cross-Correlation Analysis (DCCA) that includes partial correlation, calculated as

(3) $$\rho_{DPCCA}\left(x,y;s\right)=\frac{-C_{x,y}\left(s\right)}{\sqrt{C_{x,x}\left(s\right)\;\cdot \;C_{y,y}\left(s\right)}},$$

where C(s) is the inverted matrix ρ(s), *s* is the time scale used, and ρ is the DCCA coefficient. The DCCA coefficient is itself an extension of Detrended Cross-Correlation analysis combined with detrended fluctuation analysis (DFA), calculated as

(4) $$\rho \left(s\right)=\frac{F_{DCCA}^{2}\left(s\right)}{F_{DFA,x}\left(s\right)F_{DFA,y}\left(s\right)},$$

with detrended covariance $F_{DCCA}^{2}$,

(5) $$F_{DCCA}^{2}=\frac{\sum_{j=1}^{N-s+1}f_{DCCA}^{2}\left(s,j\right)}{N-s}$$

and detrended variance $F_{DFA,x}^{2}$,

(6) $$F_{DFA,x}^{2}=\frac{\sum_{j=1}^{N-s+1}f_{DFA,x}^{2}\left(s,j\right)}{N-s}.$$

Finally, we have

(7) $$f_{DCCA}^{2}\left(s,j\right)=\frac{\sum_{k=j}^{j+s-1}\left(X_{k}-\hat{X_{k,j}}\right)\left(Y_{k}-\hat{Y_{k,j}}\right)}{s-1}$$

and

(8) $$f_{DFA}^{2}\left(s,j\right)=\frac{\sum_{k=j}^{j+s-1}\left(X_{k}-\hat{X_{k,j}}\right)}{s-1},$$

where for a time series x, a profile $X_{t}$ is constructed as $X_{t}=\sum_{i=1}^{t}\left(x_{i}-\bar{x}\right)$ and $\hat{X_{k,j}}$ represents a linear fit of the time trend.

The calculation of the correlation between two time series capturing mussel toxicity in different production areas enables the direct analysis of the correlation between them in real time; that is, how the toxicity in one area at a given time correlates with the toxicity in the other area at the same time. This analysis is indeed important, but it can be extended to consider how the present toxicity of an area can help predict the toxicity of another area in the future. This predictive link can be further observed with different species of shellfish, toxic phytoplankton abundance, and environmental factors.

**Figure 2:**
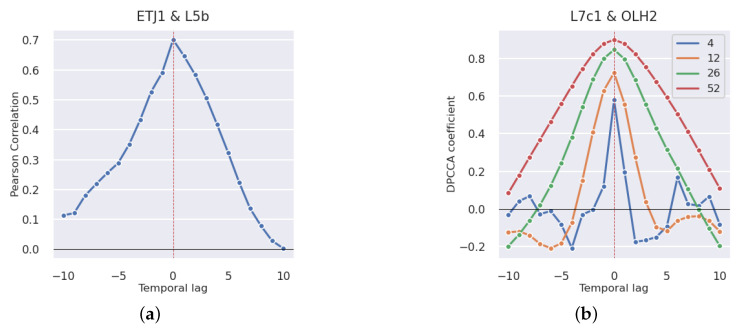
Example of time-lagged Pearson and DPCCA correlation analyses in pair of production areas. (**a**) Pearson. (**b**) DPCCA.

Knowledge about how various variables affect toxicity levels at distinct points in time strengthens the understanding of the underlying phenomena, and can also be used to predict future levels of toxicity based on other current variables. To achieve this, the correlation values between two time series were calculated with multiple time lags, from −10 weeks to +10 weeks. This calculation under the Pearson correlation with a weekly periodicity results in 21 correlation values (see Figure 2a). The DPCCA coefficient works differently due to its parameter *s*, which represents the size of the window that goes through the two time series. To avoid bias introduced by the selection of a unique *s* value, the presented charts have four different lines corresponding to 4, 12, 26, and 52 weeks, representing a month, a trimester, a semester, and a year, respectively (Figure 2b). In both figures, it is to the second area that the time lag is applied. This means that in Figure 2a, the correlation value of 0.65 at temporal lag 1 means that there is a statistically significant correlation of 0.65 between the DSP toxin concentration of a given week in area L5B and the toxin concentration of the week before in area ETJ1. The same rationale applies to plots studying the impact of different shellfish species, toxic phytoplankton abundances, and environmental conditions.

Statistical analyses were implemented using Python (version 3.9.7) and the code is publicly available on GitHub [37].

## 3. Results

### 3.1. Exploratory Analysis

**Figure 3:**
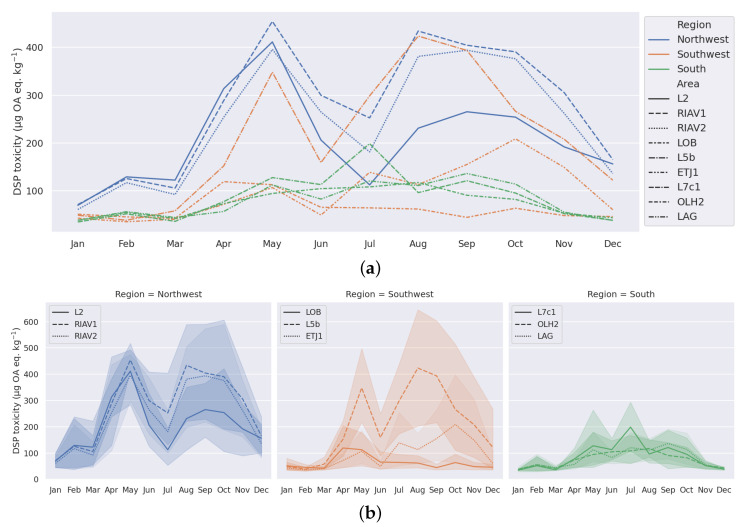
Monthly variation in DSP toxicity (mean and confidence interval) in selected mussel production areas, 2015–2020. (**a**) All regions. (**b**) By region.

**Figure 4:**
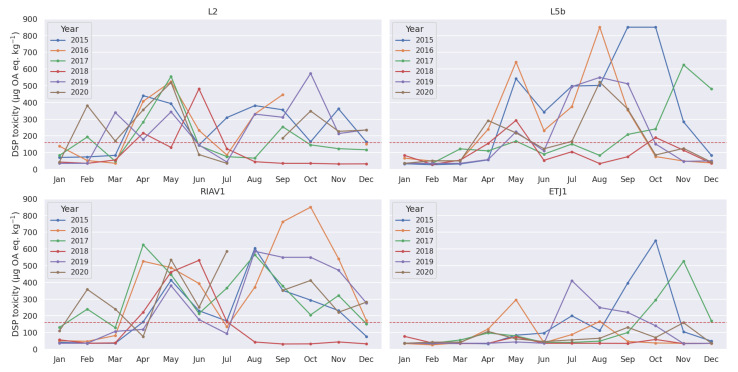
Monthly variation of DSP toxicity (mean) in selected mussel production areas by year, 2015–2020.

To help identify toxicity seasonality patterns in the data, we calculated the monthly average of the DSP toxicity concentrations in mussels from the selected production areas (e.g., Figure 3a). We can observe two distinct peaks in toxicity during the year—one in May and another between August and October. These peaks happen in the majority of the studied areas, although with different degrees of intensity across the years, as shown in Figure 4. Furthermore, we can confirm that even in areas where this seasonality pattern is more pronounced, the toxicity peaks do not occur in all the years. The year 2018, for example, has especially low values during the August–October period. The comparison of these areas in the context of their relative region on the Portuguese coast can be visualized in Figure 3b, where it is visible that as we move south, the toxicity levels in general and the intensity of the identified peaks becomes increasingly lower.

**Figure 5:**
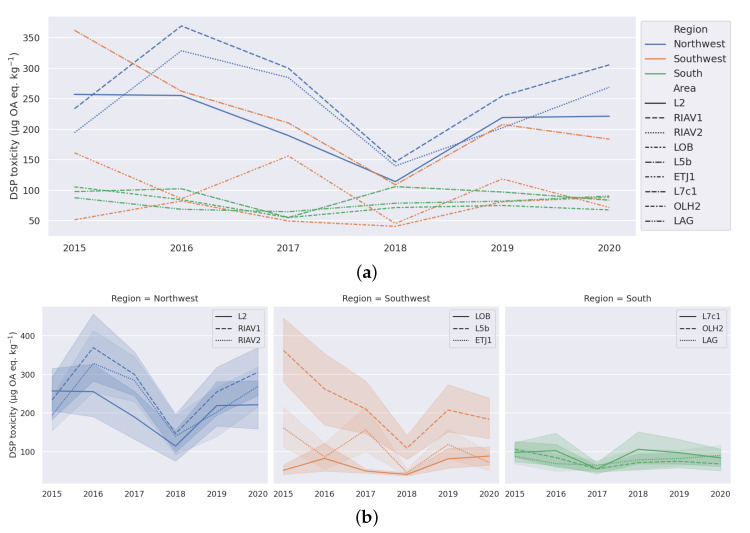
Yearly variation of DSP toxicity (mean and confidence interval) in selected mussel production areas. (**a**) All regions. (**b**) By region.

For the analysis of the trend component, a similar process was adopted and the yearly averages were plotted. Figure 5a shows lower toxicity levels in 2018 compared to preceding and succeeding years in all production areas except L7c1, OLH2, and LAG. By grouping the areas in regions, we can observe in Figure 5b that the few regions that presented minimum toxicity in 2017 instead of 2018 are all from the south region of Portugal.

**Figure 6:**
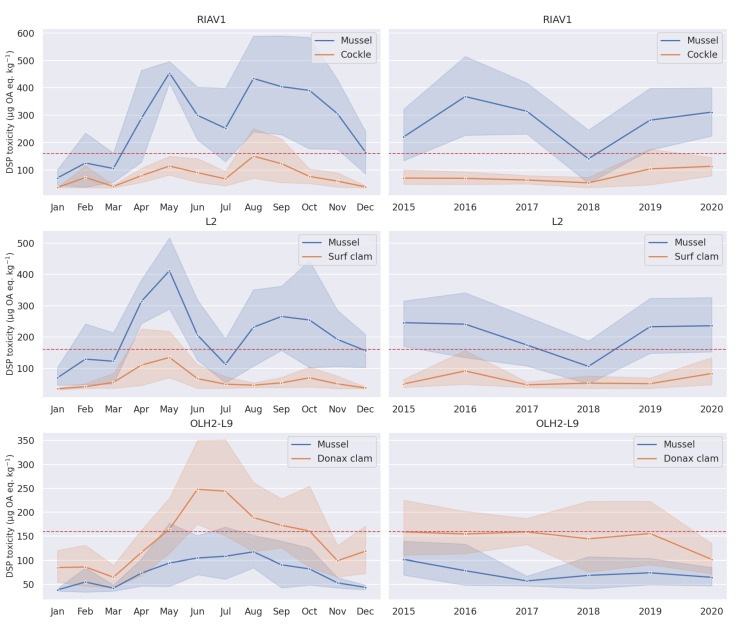
Seasonality (**left**) and trend (**right**) components of shellfish toxicity series.

We now direct our focus to the differences in seasonality and trend of toxicity in various species. Figure 6 shows a direct comparison between the seasonality component of toxicity in mussels and cockles in the RIAV1 production area, from which we can conclude that both species follow a similar seasonality pattern and that cockle has overall lower levels of toxicity. We can also see the comparison between mussel and surf clam in the L2 production area, which shows that surf clam has a less pronounced peak of toxicity between August and October. Finally, for the analysis of the relation between mussel and Donax clam, two adjacent areas were considered: OLH2 for mussel and L9 for Donax clam. We can observe in Figure 6 that Donax clam, contrary to the other analysed species, actually surpasses mussel in overall DSP toxicity and shows a single peak in its seasonality around June and July, a pattern not seen in other species.

The trend component of these time series shows identical behaviour. Mussel and cockle have a strong similarity and the latter presents overall lower values of toxicity, as seen in Figure 6. Mussel and surf clam have a weaker relation. Both show a decrease around the year 2018, but do not present any further robust similarities. Mussel and Donax show considerably different behaviours. Mussel reaches a minimum in 2017 but Donax clam has an abrupt decrease in toxicity only in 2020.

### 3.2. Correlations

#### 3.2.1. Production Areas

**Figure 7:**
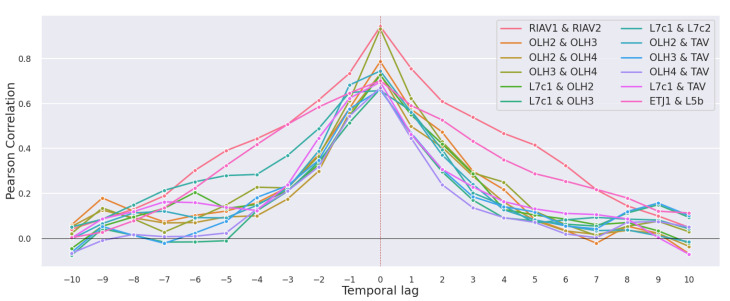
Time-lagged Pearson correlation of mussel DSP toxicity in pairs of production areas.

**Figure 8:**
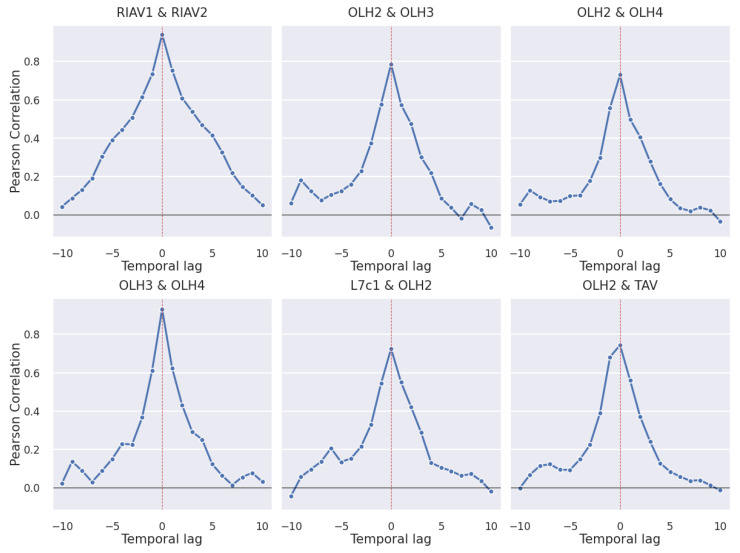
Time-lagged Pearson correlation of mussel DSP toxicity in pairs of production areas with correlation greater than 0.7 (*p*-value $<1\times 10^{-3}$ for all provided correlations).

We now present the time-lagged Pearson correlations of mussel DSP toxicity between different production areas. With the statistical significance of the results in mind, only correlations with a *p*-value < 0.05 are discussed. In Figure 7 we can see the correlation values for all the pairs of studied areas that reach a correlation of at least 0.65 at any time lag. We can observe a clear tendency for the correlation between areas to peak at time lag zero and have a symmetric curve. In Figure 8, we can see the individual pairs of production areas that have at least a 0.7 correlation. As expected, the pairs with higher correlation correspond mostly to adjacent areas, except for the pairs OLH2–TAV and L7c1–OLH2 which, although not adjacent, are still considerably close to each other.

It is also worth noticing in Figure 7 and Figure 8 that the set of areas in Ria de Aveiro, which have the prefix “RIAV”, show the highest correlations in most time lags, followed by Olhão with the prefix “OLH”.

**Figure 9:**
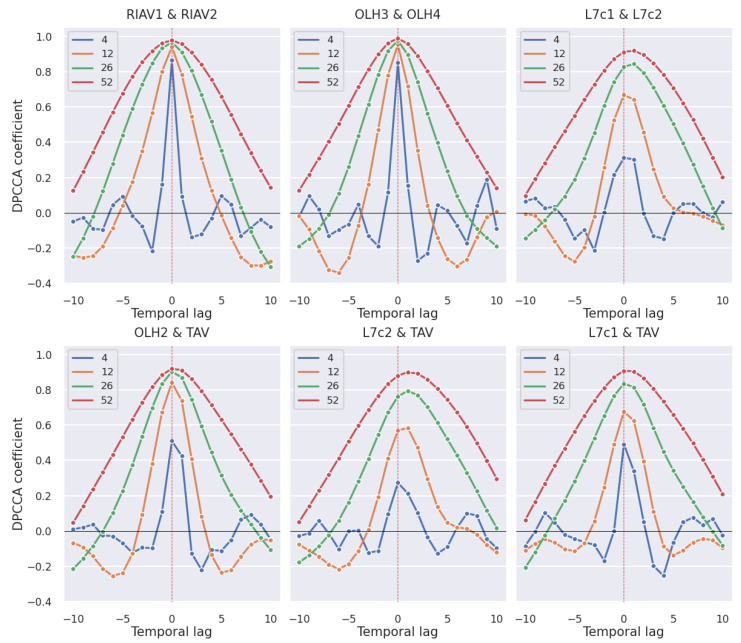
Time-lagged DPCCA of mussel DSP toxicity in pairs of production areas with window sizes of 4, 12, 26 and 52 weeks.

This correlation analysis is complemented with the calculation of the DPCCA coefficient with a varying *s* parameter, which represents the size of the sliding time window. The results were filtered with a threshold of minimum coefficient equal to 0.9 and are shown in Figure 9, where we can observe that the areas in the Ria de Aveiro (RIAV) and Olhão (OLH) regions present the highest correlations. It is worth noticing that we have more pairs of non-adjacent areas selected compared with the Pearson correlation analysis. In addition, there are two pairs, L7c2–TAV and L7c1–L7c2, that peak at lag +1 and not lag 0. As expected, the greater the *s* parameter, the smoother and greater the correlation. Still, some of the most correlated areas also present considerably high values with lower *s* values, indicating robustness in the obtained results.

#### 3.2.2. Shellfish Species

We now complement our analysis with the study of how different species behave in the same conditions, i.e., how two shellfish species in the same production area relate to each other in their toxicity levels.

**Figure 10:**
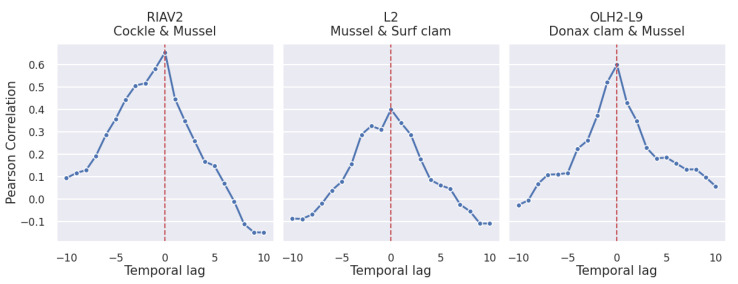
Time-lagged Pearson correlation of DSP toxicity in pairs of shellfish species (*p*-value $<1\times 10^{-3}$).

By analysing the results in Figure 10, we can conclude that the correlations between the same species in different areas tend to be stronger than the correlation between different species in the same area. However, we can still observe correlations as high as 0.6. Furthermore, contrary to the previous correlation graphics, we now have asymmetric curves. This indicates that the DSP toxicity of a given shellfish species can be more useful at predicting the toxicity of a co-occurring shellfish species than the contrary. The asymmetric relation can be explained by the fact that some species accumulate toxins faster than others, which results in a faster increase in toxicity levels despite being subjected to the same conditions. Based on Figure 10 we can conclude that the strongest correlations are between mussel and cockle and between mussel and Donax clam, while the pair of surf clam and mussel achieves a maximum correlation of 0.4 at time lag 0.

**Figure 11:**
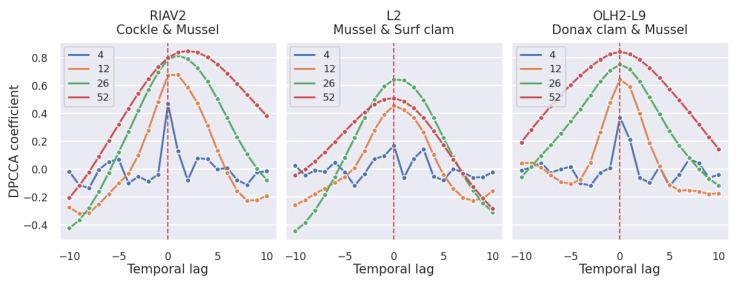
Time-lagged DPCCA of DSP toxicity in pairs of shellfish species with window sizes of 4, 12, 26 and 52 weeks.

The asymmetry seen in the curves of the Pearson correlation is confirmed by the curves obtained through the calculation of the DPCCA coefficient, as shown in Figure 11.

#### 3.2.3. Toxic Phytoplankton Abundances

**Figure 12:**
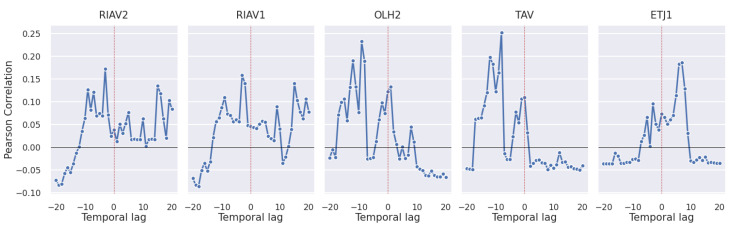
Time-lagged Pearson correlation between DSP toxicity in mussel and toxic phytoplankton abundances.

The previous analysis is now extended to the time-lagged correlations between toxic phytoplankton abundances and the toxicity of mussels. Since the toxicity present in shellfish originates from the filtering of phytoplankton, we expected these two variables to be robustly correlated. Yet, as we can see in Figure 12, this is not the case. To ensure that the lower correlation values are not due to higher times of toxin accumulation, the time lag interval was increased to −20 to 20 weeks; however, the correlations persisted with relatively low values.

#### 3.2.4. Oceanographic and Meteorological Conditions

**Figure 13:**
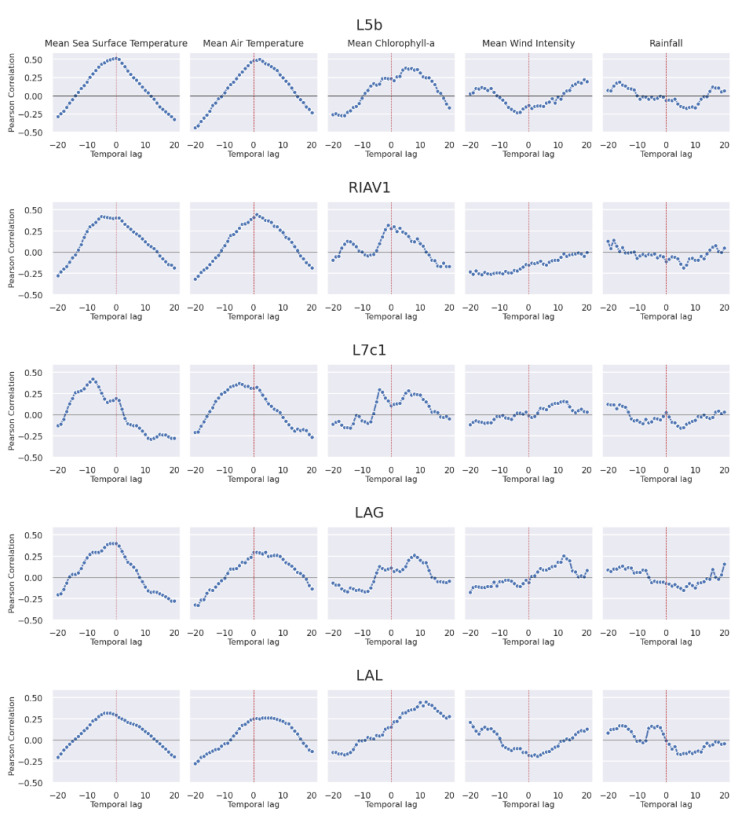
Time-lagged Pearson correlation between DSP toxicity in mussel and oceanographic and meteorological conditions.

Finally, we look at the impact of various oceanographic and meteorological conditions on the DSP toxicity in shellfish. The correlation values shown in Figure 13 correspond to the time-lagged Pearson correlation between the stated variables on top and the mussels’ DSP toxicity in the stated production area. The variables under analysis are described in Table 1c and correspond to: (i) mean sea surface temperature; (ii) mean air temperature; (iii) mean chlorophyll-a concentration; (iv) mean wind intensity; and (v) accumulated rainfall.

The obtained correlations achieve relatively high values, with the air and sea surface temperature reaching a correlation as high as 0.5. The shape of the obtained curves is also worth studying. The mean sea surface temperature bell-like curve shows a tendency to increase faster than when decreasing, with some cases in which its peak is to the left of time lag 0. This behaviour is more pronounced in the south, namely in the production areas L7c1 and LAG. Air temperature presents a curve more centred at time lag 0 and no consistent skewing direction. Chlorophyll-a shows a less well-defined correlation curve but with a clear tendency to peak to the right of lag 0, highlighting it as a possible predictor of DSP toxin levels. Finally, both wind intensity and rainfall show correlations below 0.2 and no consistent curve shape.

## 4. Discussion

### 4.1. Seasonality-Trend Analysis

Through the analysis of time series of DSP toxicity, we were able to identify multiple interesting patterns in its components. The DSP toxicity in the majority of production areas under analysis follows a well-defined seasonality, with peaks of toxicity in May and between August and October (Figure 3a). An analysis of marine biotoxins in shellfish across the Portuguese coast between 1986 and 2006 reached similar results, indicating that the DSP season starts around May and in some years can be slightly delayed to August [24]. These results are consistent with our analysis and strengthen our conclusion about the seasonality of DSP toxicity on the Portuguese coast.

An additional relevant result is the common trend of an overall decrease in toxicity between 2015 and 2018, followed by an increase in toxicity until 2020 (Figure 5a).

We also identified relations between geographical location and overall level of toxicity. Our analysis shows that the production areas in the south region present the lowest levels of toxicity, while the north west region has noticeably higher levels of toxicity (Figure 3b and Figure 5b). The work by Vale et al. [24] also points out this relation between DSP toxicity and the geographical location along the Portuguese coast, corroborating the existence of this pattern since 1986.

We complement our analysis with the comparison of toxicity seasonality and trend patterns between species of shellfish (Figure 6). Mussel presents higher DSP toxicity when compared with cockle and surf clam, but overall lower concentrations than Donax clam. This is explained by the slower elimination of dinophysistoxin-2, especially in its free form, by both mussel and Donax clam [38]. The increased levels of toxicity in mussel, when compared to other species, have been described in other works. The work by Vale and Sampayo [39] found mussel and cockle contained the highest DSP toxicity when compared to peppery furrow shell, carpet shell, oyster, razor clam, and clam. Works by Wu et al. [40] and Lee et al. [41] also found mussel had a higher accumulation of DSP toxins when compared to oyster (*M. gigas*). Despite considerable different levels of toxicity between the species under analysis, the seasonality patterns are consistent inside each production area. Furthermore, Mussel and Donax clam show a distinct pattern with a single peak between June and August in OLH2-L9.

### 4.2. Correlation Study

By calculating time-lagged correlations between the toxicity series in pairs of areas, we were able to identify geographical correlates. There is a clear tendency for the correlation between production areas to peak at time lag 0, i.e., when both time series are aligned (Figure 8). This indicates an inclination of the toxicity values to vary simultaneously in geographically close production areas. We further highlight that in some cases, especially in adjacent areas, the correlations are high enough in time lags ±2 and ±1 to hypothesize the use of one production area to make predictions about the development of toxicity in another area. This would contribute to better informing the shellfisheries sector and help manage production and distribution into the market.

The analysis of correlations between toxicity values was extended to an inter-species setting, where we calculated the correlations between pairs of shellfish species in the same location. The obtained results (Figure 10 and Figure 11) show that the correlations between species are weaker than the ones between the same species but in different locations. Still, we have high enough correlations to conclude that a strong relationship exists between these species, especially between mussel and two others: cockle and Donax clam. It is also worth noticing that, contrary to the correlations seen between production areas, these plots do not show a symmetric curve, which indicates that one of the analysed species is more useful at predicting the other due to a faster accumulation of toxins. An example of this is seen in Figure 10, where we can observe that cockle should be better at predicting mussel than the other way around.

An additional important relation to study is the one between shellfish toxicity and toxic phytoplankton cell counts. Our analysis indicates a lower correlation than expected, since the toxins that accumulate in shellfish originate from the phytoplankton that these species consume. Still, although low, a correlation between toxicity levels in phytoplankton and shellfish does exist [13,42] and can be used as complementary information to make predictions based on other variables with higher correlations, such as shellfish toxicity in adjacent areas and other species.

Finally, the correlation analysis of the impact of oceanographic and meteorological conditions on the toxicity levels of shellfish highlighted relevant patterns (Figure 13). Rainfall can influence the values of DSP toxicity by leading to river drainage, as shown in the data of the Portuguese coast studied by Vale and Sampayo [43] that indicate more frequent surpassing of the safety levels of DSP toxicity in the months with the lowest historical average rainfall. Wind stress can also harm the ideal conditions for the proliferation of toxin-producing phytoplankton [44]. Still, neither of these variables showed strong correlations with the levels of DSP toxicity, possibly due to the mentioned phenomena occurring mainly at locations with specific geographical characteristics that were not present in the production areas under analysis. Regarding temperature, available knowledge of the relationships between temperature and DSP toxicity levels indicates that a positive correlation exists until a certain ideal water temperature is reached [15]. It is precisely the sea surface temperature that shows one of the highest correlations with DSP toxicity, obtaining similarly high values to air temperature. Both reach their highest correlations mainly at around time lag 0. Air temperature presents no consistent skew of its curve, and the sea surface temperature shows higher correlation values in the negative time lags. The final variable under analysis, chlorophyll-a concentration, presents its higher correlation, 0.4, at around time lag +10, a behaviour not found in the other variables.

## 5. Conclusions

In our study, we used data sources of DSP toxicity in shellfish, toxic phytoplankton cell counts, and environmental conditions across the Portuguese coast to identify relevant patterns of shellfish toxicity and analyse relationships against various biotic and abiotic contextual variables. Our results improve the understanding of DSP toxicity dynamics in shellfish and show how this type of analysis can help extend the knowledge about associations in the shellfish’s ecosystem and their impact on shellfish DSP toxicity. We further motivate the development of a shellfish safety forecasting system capable of aiding in the monitoring process of shellfish production.

In particular, we explored DSP toxicity patterns across geographical locations and shellfish species. We identified a tendency in seasonality to reach peaks of toxicity in May and between August and October, an overall decrease in toxicity from 2015 to 2018, and reduced toxicity in the South region. The latter pattern corroborates results previously obtained on the Portuguese coast.

Additionally, we studied the relation between DSP toxicity and geographical location, shellfish species, toxic phytoplankton abundance, and environmental conditions. Through the introduction of temporal lags in the correlation analysis, we were able to identify regions, such as Ria de Aveiro (RIAV) and Olhão (OLH), where the correlations are considerably strong, and therefore represent adequate candidates for a predictive analysis of DSP toxicity. The employed correlation analysis further reveals a strong relation between DSP toxicity in mussel and both cockle and Donax clam.

The inclusion of additional contextual variables with a possible association with DSP toxicity, such as lux readings [45], is highlighted as a complementary effort to strengthen the predictive accuracy of the envisioned system. Our work has helped identify possible highly correlated variables that can support variable selection and model building. Therefore, as possible future work, we highlight the development of a forecasting system to predict shellfish contamination based on a state-of-the-art machine learning methods (as reviewed by Cruz et al. [16]). This system would consider as variables the DSP toxicity of adjacent areas and highly correlated shellfish species, complemented by information about toxic phytoplankton abundances, oceanographic conditions such as water temperature, and additional variables associated with DSP toxicity.

## Figures and Tables

**Table 1:** Variable descriptions for the used datasets.

(**a**) Diarrhetic Shellfish Poisoning (DSP) toxins dataset.				
	**Description**	**Range**	**Mean**	**Type**
**Date**	Date of the measurement	2015-01-05 to 2020-12-29	-	Date
**Production Area**	Production area, as defined by IPMA, where the measurement was performed	-	-	Categorical
**Species**	Species analysed	-	-	Categorical
**DSP Toxins**	Concentration of Diarrhetic Shellfish Poisoning toxins (μg OA eq. kg^−1^)	9 to 1945	110.58	Numerical
(**b**) Toxic phytoplankton cell counts dataset.				
	**Description**	**Range**	**Mean**	**Type**
**Date**	Date of the measurement	2015-01-05 to 2020-12-29	-	Date
**Production Area**	Production area, as defined by IPMA, where the measurement was performed	-	-	Categorical
**DSP Toxins Producers**	Toxic phytoplankton abundances (cell/L)	20 to 1907840	1772.27	Numerical
(**c**) Oceanographic and meteorological dataset.				
	**Description**	**Range**	**Mean**	**Type**
**Date**	Date of the measurement	2015-01-05 to 2020-12-29	-	Date
**Production Area**	Production area, as defined by IPMA, where the measurement was performed	-	-	Categorical
**Mean SST**	Weekly mean Sea Surface Temperature (SST) obtained from Copernicus (Kelvin)	285 to 297	290.08	Numerical
**Mean Chlorophyll-a**	Weekly mean of chlorophyll-a concentration obtained from Copernicus (mg/L)	0.23 to 27.25	3.74	Numerical
**Mean Air Temperature**	Weekly mean air temperature (Celsius) at 1.5 m of altitude	3.93 to 28.69	16.34	Numerical
**Mean Wind Intensity**	Weekly mean wind speed (km/h)	0.33 to 8.75	2.82	Numerical
**Rainfall**	Weekly mean of accumulated precipitation (millimeters)	0 to 24.59	1.57	Numerical

## Data Availability

Publicly available datasets were analysed in this study—these are not yet provided to comply with double blind reviewing.
